# Concept development of an on-chip PET system

**DOI:** 10.1186/s40658-022-00467-x

**Published:** 2022-05-19

**Authors:** Christoph Clement, Gabriele Birindelli, Marco Pizzichemi, Fiammetta Pagano, Marianna Kruithof-De Julio, Sibylle Ziegler, Axel Rominger, Etiennette Auffray, Kuangyu Shi

**Affiliations:** 1 Department of Nuclear Medicine, Inselspital Bern, University of Bern, Bern, Switzerland; 2 EP Department, CERN, Geneva, Switzerland; 3 Physics Department, University of Milano-Bicocca, Milan, Italy; 4 Department for Biomedical Research, Inselspital Bern, University of Bern, Bern, Switzerland; 5 Department of Nuclear Medicine, University Hospital, LMU Munich, Munich, Germany

**Keywords:** CNN, Deep learning, GATE, Monte-Carlo simulation, Organs-on-chips, PET, Reconstruction, SART

## Abstract

**Background:**

Organs-on-Chips (OOCs), microdevices mimicking in vivo organs, find growing applications in disease modeling and drug discovery. With the increasing number of uses comes a strong demand for imaging capabilities of OOCs as monitoring physiologic processes within OOCs is vital for the continuous improvement of this technology. Positron Emission Tomography (PET) would be ideal for OOC imaging, however, current PET systems are insufficient for this task due to their inadequate spatial resolution. In this work, we propose the concept of an On-Chip PET system capable of imaging OOCs and optimize its design using a Monte Carlo Simulation (MCS).

**Material and methods:**

The proposed system consists of four detectors arranged around the OOC device. Each detector is made of two monolithic LYSO crystals and covered with Silicon photomultipliers (SiPMs) on multiple surfaces. We use a Convolutional Neural Network (CNN) trained with data from a MCS to predict the first gamma-ray interaction position inside the detector from the light patterns that are recorded by the SiPMs on the detector’s surfaces.

**Results:**

The CNN achieves a mean average prediction error of 0.80 mm in the best configuration. The proposed system achieves a sensitivity of 34.81% for 13 mm thick crystals and does not show a prediction degradation near the boundaries of the detector. We use the trained network to reconstruct an image of a grid of 21 point sources spread across the field-of-view and obtain a mean spatial resolution of 0.55 mm. We show that 25,000 Line of Responses (LORs) are needed to reconstruct a realistic OOC phantom with adequate image quality.

**Conclusions:**

We demonstrate that it is possible to achieve a spatial resolution of almost 0.5 mm in a PET system made of multiple monolithic LYSO crystals by directly predicting the scintillation position from light patterns created with SiPMs. We observe that a thinner crystal performs better than a thicker one, that increasing the SiPM size from 3 mm to 6 mm only slightly decreases the prediction performance, and that certain surfaces encode significantly more information for the scintillation-point prediction than others.

## Background

Organs-on-Chips (OOCs) are microdevices that mimic in vivo organs. They contain 3D tissue cultures connected by microfluidic channels that add biomechanical forces to the system [1]. OOCs have sparked the interest of researchers in the past decade, especially in the drug discovery and development process, as they can enhance several steps in this process [2].

With the growing number of use cases for OOCs comes an increasing demand for novel measurement capabilities. Monitoring metabolism or other physiologic and pathophysiologic processes in OOCs is critical to refining the technology to closely resemble in vivo physiology and promote its application in new biological models.

Positron Emission Tomography (PET) would be the ideal candidate for OOC imaging due to its ability to retrieve in vivo information about metabolism and molecular pathways [3]. However, current imaging devices for measuring PET tracer uptake in either small animals or cell cultures are inadequate for the task of OOC imaging due to their limited spatial resolution [4]. Several degrading factors limit PET systems’ spatial resolution, such as the distance that a positron travels before annihilating with an electron, scattering of the emerging gamma-rays in the tissue, and the detector’s resolution.

In recent years, there has been a trend in pre-clinical PET research toward using monolithic instead of pixelated crystals as detectors to increase the spatial resolution. The resolution in monolithic crystals is not inherently limited by the pixel size in contrast to pixelated detectors but can be improved with more advanced readout schemes and data processing methods. The key to increasing the resolution is to predict the first gamma-ray interaction position in the detector as precisely as possible and thus improve the estimation of the Line of Responses (LORs) [5].

In literature, several works have tackled the problem of predicting the gamma-ray interaction position in monolithic crystals with either analytical or data-driven approaches.

Clement et al. [6] (not related to the first author of this work) implemented one of the earliest Neural Network (NN) based methods to predict the Depth of Interaction (DOI) in monolithic crystals. They used signals of solid-state photodetectors that fully cover the crystal as input for three multilayer NNs. Each NN predicts two of the three coordinates of the gamma-ray interaction position. The authors set up a Monte Carlo Simulation (MCS) to generate training data for the networks and demonstrated that the NN-based approach yields better results than a baseline method using Anger logic with a Full Width at Half Maximum (FWHM) of 2.0 mm compared to 3.5 mm.

The work by Wang et al. [7] introduced a monolithic PET detector system that can estimate gamma-ray interaction positions with NNs. They trained one network to estimate the plane position and another to predict the DOI. The input data were created with a simplified readout scheme with signals from a Photomultiplier Tube (PMT) on one side of the crystal. The system achieves spatial and DOI resolutions of 2.0 mm.

Marcinkowski et al. [8] investigated a high-resolution small-animal PET system based on a continuous crystal. They coupled a Lutetium-yttrium oxyorthosilicate (LYSO) crystal with a Digital Photon Counter and determined the gamma-ray interaction position using mean-nearest-neighbor positioning. The system reaches a spatial FWHM of 0.60 mm and a DOI FWHM of 1.66 mm.

The work by Tao et al. [9] compared four different NN architectures that estimate gamma-ray interaction positions in monolithic crystals. They trained fully connected and Convolutional Neural Networks (CNNs) with regression and classification heads with mean detector response functions as input. The different networks reached prediction errors between 2.0 mm and 2.6 mm. The authors found that deep learning methods reduce the memory cost by a factor of one to two orders of magnitude compared to searching-based methods.

Sanaat and Zaidi [10] presented another approach to estimate the DOI in a monolithic crystal using a NN. They trained a multilayer perceptron that outputs the 3D gamma-ray interaction position with data from an MCS. Their proposed approach reaches a spatial resolution of 1.54 mm in the *x*-*y* plane, which is better than an Anger logic-based method.

Decuyper et al. [11] simulated the interaction of a gamma-ray grid with a monolithic LYSO crystal. With the simulated data, they trained a NN that estimates the first gamma-ray interaction position. The optimal amount of training data and network design was determined to overcome the problem of overfitting. With the NN, they achieved a median positioning error of 0.77 mm and a 2D FWHM of 0.46 mm, which was an improvement of 17% compared to a nearest-neighbor algorithm. The prediction performance was even more improved when only using non-Compton scattered events.

Jaliparthi et al. [12] developed AnnPET, a monolithic annular PET system consisting of a single annulus-shaped LYSO crystal with Silicon photomultiplier (SiPM) arrays attached to its outer surfaces. They employed a ten-layer CNN to estimate the gamma-ray interaction position and reached single dimension Mean Absolute Error (MAE) values between 0.42 mm and 0.54 mm for the position prediction. When using the trained network for reconstruction, they achieved FWHM values between 0.71 and 0.80 mm, which are around 0.4 mm better than the results of a center-of-mass algorithm.

The work by Liu et al. [13] introduces a dedicated PET scanner for microfluidic radiobioassays that is made up of two detector panels placed above and below the microfluidic chip. The system achieves a spatial resolution of around 1.8 mm and the authors demonstrate its capability to image cellular pharmacokinetics.

In this work, we propose an On-Chip PET system to make functional imaging of OOCs possible. The novelties presented in this work are twofold. First, we design a scanner made up of four detectors that consist of two glued-together monolithic crystals each. Second, we train a CNN directly with the light pattern images that emerge on the surfaces of the detectors to predict the first scintillation positions inside the detectors. We optimize the design of the system with an MCS to create datasets of light pattern images emerging on the surfaces of the detectors through scintillation. With these datasets, we train and evaluate CNNs that predict the first interaction positions of the gamma rays inside the detector. With the predicted scintillation positions, we reconstruct the insides of the detector using Simultaneous Algebraic Reconstruction Technique (SART) [14].

Our proposed system would support two important applications of OOCs in preclinical use - disease modeling and precision medicine [15]. Measuring cellular pharmacokinetics helps to understand human diseases by modeling biochemical and genetic manipulations. The analysis of patient-derived organoids enables finding the most suitable drug on a per-patient basis.

## Methods

### Monte Carlo simulation

We model the interaction of the proposed system with a Fluorine-18 positron source in an MCS built with the Geant4 Application for Emission Tomography (GATE) tool [16–18]. GATE enables the creation of MCSs in the field of nuclear medicine through a macro language that controls the experimental settings. It is built as a wrapper around the Geant4 simulation toolkit that enables simulating ”the passage of particles through matter” for a wide range of physics processes, particles, and materials over a broad energy spectrum [19–21].

A GATE simulation consists of the following parts that are described in more detail in the next sections: a scanner geometry, a phantom, material properties, physics processes, boundary surfaces, and a primary particle source.

#### Geometry

In GATE, the concept of a *system* plays a crucial role if one wants to store information about particles and physical processes in the simulation. A system is defined as a template for predefined scanner types with specific geometries. Different geometrical shapes are organized in a tree-level structure for these scanners to build up the final geometry.

In this work, we use the most generic system in GATE, the *scanner*. In our case, the scanner is defined as a box-shaped volume and placed in the world volume. The purpose of the scanner volume is to encapsulate our proposed PET system. The box-shaped detector volume is placed inside the scanner volume and repeated four times with a ring repeater around the *z*-axis. Inside the detector volume, the box-shaped crystal volume is placed and repeated two times with a linear repeater. Table 1 contains the lengths, translations, and materials of each volume. We evaluate two different crystal thicknesses, 13 mm and 26 mm. All dimensions of the crystals are chosen such that arrays of commercially available SiPMs fit on the surfaces. The properties of the respective materials of the different volumes are described in the next section.

**Table 1:** Geometry setup of the GATE simulation

Name	Parent volume	Type	Lengths [mm]	Material	Repeater
World	–	Box	126.3	Vacuum	–
Scanner	World	Box	114.8	Vacuum	–
Detector	Scanner	Box	52.2, 13.1 or 26.1, 104.4	Epoxy	Ring 4
Crystal	Detector	Box	52.0, 13.0 or 26.0, 52.0	LYSO	Linear 2

If only one length is given, it is used for all dimensions. In the Repeater column, the repeater type is followed by the number of repeats

In GATE, it is important to attach the created volumes to the system to be able to record hits in them. In our case, the detector volume is attached to the first level of the scanner system and the crystal volume to the second one. To both of these volumes, we attach a crystal sensitive detector that records the hits in them.

**Fig. 1:**
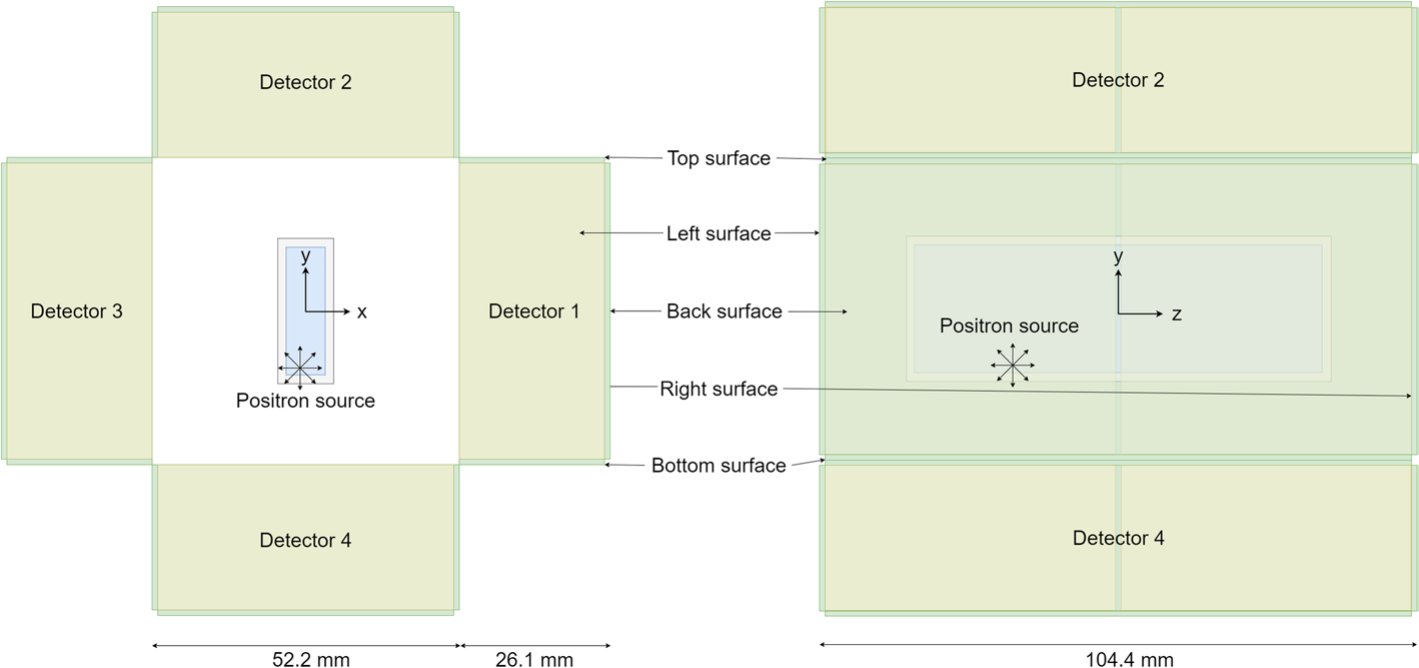
Simulation setup viewed from the front and the side. The yellow volumes represent the crystals, the green ones the epoxy layer around the crystal, and the blue one the volume from which the source position is sampled to create the training dataset

Figure 1 depicts the simulation setup viewed from the front and the side.

#### Phantoms

We use different types of phantoms depending on the purpose of the dataset that is created by the simulation.

For training, we use a simplified model of an OOC device consisting of a box-shaped water volume surrounded by a PMMA box that is placed in the middle of the detector. The PMMA box has side lengths of 10 mm $\times$ 26 mmm $\times$ 76 mm while those of the water box are 4 mm shorter each.

For evaluating the sensitivity of the system, we instead add a 1 mm thick and 104 mm long water cylinder to the simulation that is placed along the *z*-axis of the scanner.

To demonstrate that our system is able to capture the small volumes found in OOC devices, we create a more complex OOC phantom consisting of the PMMA box mentioned above that contains four water spheres with a radius of 2 mm each, which are distributed along the *z*-axis.

In “Sources” section, the sources that are combined with each of the phantoms are described.

#### Materials

The materials of the world and scanner volumes are set to vacuum, the detector’s to epoxy, and the crystal’s to LYSO. In this way, one detector consists of two LYSO crystals that are surrounded by a 0.1 mm thick layer of epoxy, which acts as the glue and optical medium between the crystals and SiPMs.

In Table 2, the material properties are described, and in Table 3, the scintillation properties of LYSO are shown.

**Table 2:** Properties of the materials used in the GATE simulation

Property	Epoxy	LYSO	Vacuum	PMMA	Water
Composition	C (n = 1),	Lu (f = 0.71),	H (n = 1)	H (f = 0.08),	H (n = 2),
	H (n = 1),	Y (f = 0.04),		C (f = 0.60),	O (n = 1)
	O (n = 1)	Si (f = 0.64),		O (f = 0.32)	
		O (f = 0.18)			
Density [g/cm^3^]	1.0	7.36	0.00	1.19	1.00
State	Solid	Solid	–	Solid	Liquid
Refractive Index	1.5	1.80–1.88	1.0	1.49	1.33
Absorption Length	50 m	1–438 mm	50 m	50 m	50 m

If a range is given, the property is energy dependent

**Table 3:** Scintillation properties of LYSO

Property	Value
Scintillation Yield [1/Mev]	40,000
Resolution Scale	4.8
Fast Time Constant [ns]	36
Yield Ratio	1
Fast Component	0–1.699

If a range is given, the property is energy dependent

#### Physics and cuts

As a physics list, the Electromagnetics (EM) constructor with option four is chosen, which uses the most accurate standard and low-energy models available in Geant4. Table 4 shows the enabled physics processes with their selected models. We set a cut of 0.1 mm for gammas, electrons, and positrons in the scanner volume.

**Table 4:** Added processes in the GATE simulation

Name	Model
Optical Absorption	Standard
Optical Boundary	Standard
Scintillation	Standard
Photo Electric	Standard
Compton	Standard Model
Rayleigh Scattering	Penelope
Electron Ionisation	Standard e+ & e-
Positron Annihilation	Standard
Bremssstrahlung	Standard e+ & e-
Multiple Scattering e+ & e-	Standard

#### Surfaces

We use Geant4’s *unified* model to define the surfaces in the simulation. The surfaces between the detector and crystal volumes are *dielectric-dielectric* ones with a *ground* finish and a *sigmaalpha* value of 0.01 corresponding to a typical polished crystal. Their specular lobe constant is set to 1.0.

To detect optical photons in GATE, it is necessary to use *dielectric-metal* boundaries. As we want to detect the optical photons that are leaving the Epoxy layer and thus entering the SiPMs, we add *dielectric-metal* surfaces to the boundaries between the scanner and detector volumes. These surfaces have specular lobe, specular spike, and backscatter constants of 0.0, a reflectivity of 0.0, and an efficiency of 1.0.

#### Sources

To create the training and testing datasets, we add a source that emits Fluorine-18 positrons with an activity of 1000 Bq to the simulation setup. The position of the source is sampled from the box-shaped water volume described in “Phantoms” section.

To evaluate the spatial resolution of the system, we create another dataset where 21 F18-point sources with a total activity of 1000 Bq are arranged in a 7 $\times$ 3 grid with a distance of 10 mm in between each source, which are placed in the box-shaped water volume.

A 104 mm long line source is placed along the *z*-axis inside the water cylinder to determine the sensitivity of the system.

To create the more complex OOC phantom, we place four hot sphere-shaped sources with radii of 0.4 mm, 0.5 mm, 0.6 mm, and 0.7 mm inside the four water spheres described in “Phantoms” section. These hot sources each have an activity concentration of 1000 Bq/mm$^3$, and are surrounded by cold sphere-shaped sources with a radius of 2 mm and an activity concentration of 100 Bq/mm$^3$.

### Dataset creation

In the first step, we run the GATE simulation described in the previous section in parallel. In the second step, we post-process the hits output files from the GATE simulation runs. For each primary event, in which one positron is emitted, zero to two samples of the training dataset are created. The amount of created samples depends on the number of detectors in which the gamma-rays interact. If there is a scintillation event in at least one detector, the position of the first interaction of the gamma-ray as well as the corresponding light patterns that emerge on the surfaces of the detectors, are saved. To create the light patterns, we simulate the SiPMs that are attached to the surfaces of the detector. This procedure is described in more detail in the next section. Table 5 gives an overview of the created datasets. Depending on how many different datasets are needed to evaluate a design choice, we use either the smaller (100k) or larger (1M) versions.

**Table 5:** Overview of created datasets

Crystal thickness [mm]	Source	Length	SiPM sizes [mm]	Purpose
13 mm	Box	100k	3, 4, 6	Training
13 mm	Box	10k	3, 4, 6	Testing
26 mm	Box	100k	3, 4, 6	Training
26 mm	Box	10k	3, 4, 6	Testing
13 mm	Box	1M	3	Training
13 mm	Line	10k	3	Sensitivity
26 mm	Line	10k	3	Sensitivity
13 mm	Point-Sources Grid	1M	3	Reconstruction
13 mm	Spheres	300k	3	OOC imaging capability

If multiple SiPM sizes are given, three separate versions of the dataset exist. Otherwise, one row corresponds to one dataset

### Light pattern creation

To create the light patterns that emerge on the surfaces of the detector, we simulate the SiPMs that cover the surfaces of the detector with SimSiPM, a C++ library for SiPM simulation [22]. We want to evaluate the performance of our proposed system with three different options for the size of the photosensitive area of the SiPMs. The SiPMs simulated in this work come from Hamamatsu’s S14161 series with photosensitive area sizes of either 3, 4, or 6 mm [23]. From now on, we refer to the SiPMs as small (3 mm), medium (4 mm), and large (6 mm). The properties of the SiPMs are described in Table 6.

**Table 6:** Properties of the simulated Hamamatsu SiPMs [23]

Property	S14160-3050HS	S14160-4050HS	S14160-6050HS
Photosensitive area/channel [mm]	3.0 $\times$ 3.0	4.0 $\times$ 4.0	6.0 $\times$ 6.0
Pixels per Channel	3531	6331	14331
Pixel Pitch [µm]		50	
Photon Detection Efficiency [% at 450 nm]		50	
Fill Factor [%]		74	
Rise Time [ns]		2	
Fall Time [ns]		190	
Recovery Time [ns]		50	
Dark Count Rate [kHz/mm$^2$]		110	
Crosstalk Probability [%]		7	
Afterpulse Probability [%]		6	

The light patterns are created with the following procedure:

1. Assign the corresponding SiPM to each photon leaving the epoxy layer depending on the photon’s position.
2. Add photon times for each SiPM on each surface.
3. Integrate over the SiPM signal to compute the output value of each SiPM on each surface.
4. Pad the resulting light patterns with zeros such that they all have the same shape. This step is necessary to be able to use the stacked light patterns as input to the CNN.

**Fig. 2:**
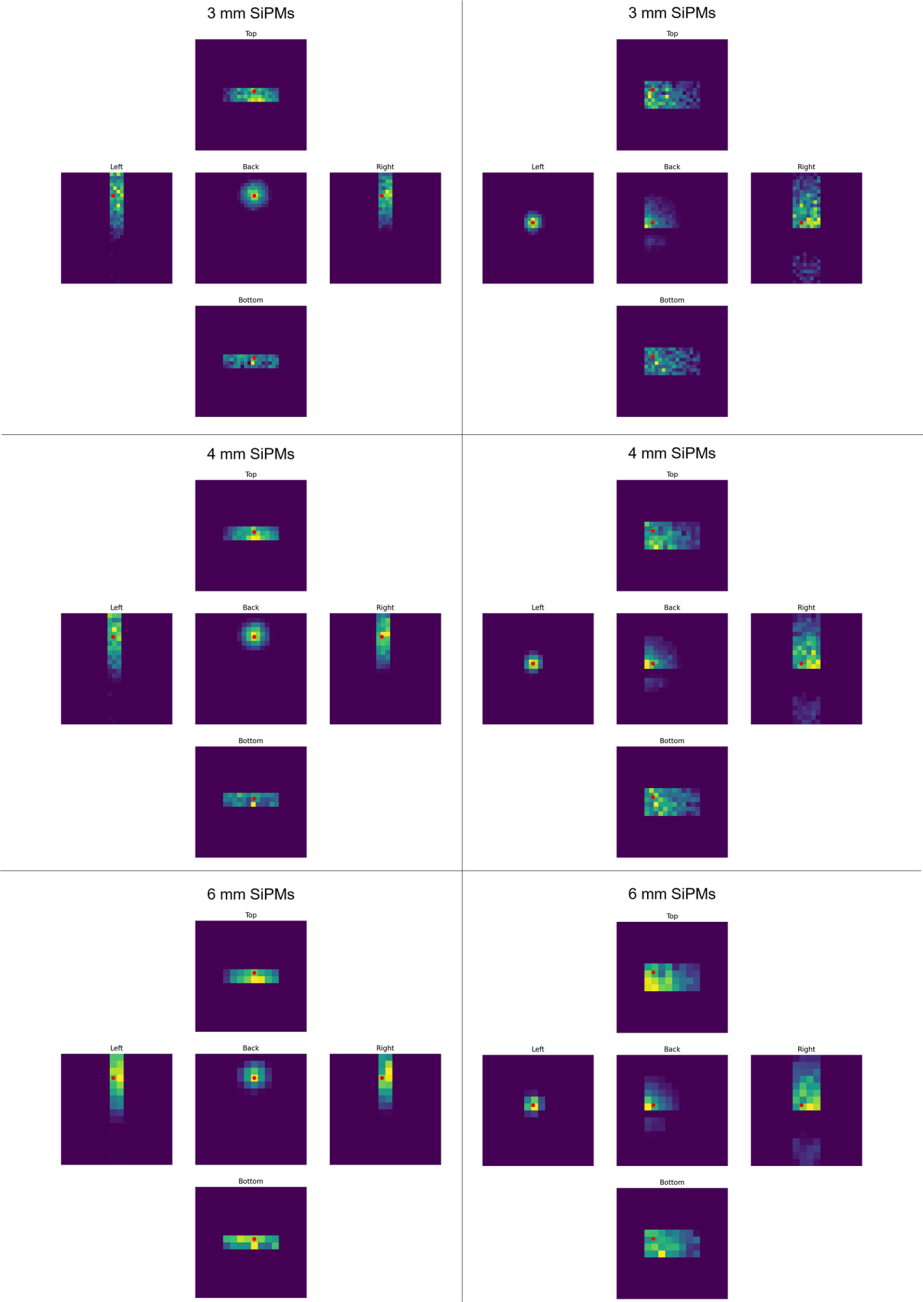
Light patterns recorded with different SiPMs sizes of 13 mm thick crystals (left) and 26 mm thick crystals (right). The light patterns recorded with the SiPMs on all surfaces of the detector except the front one are shown. The top light patterns are created with a photosensitive area size of the SiPMs of 3 mm, the middle ones with 4 mm, and the bottom ones with 6 mm. The light patterns are padded such that they all have the same square shape. The red dot is the scintillation position of the gamma-ray inside the crystal projected onto each surface

Six light patterns recorded with different SiPMs sizes of 13 mm and 26 mm thick crystals are depicted in Fig. 2.

### Scintillation position prediction

With the created dataset described in the previous section, we train a CNN that predicts the gamma-ray interaction position inside the crystal. The input to the network is the stacked light patterns recorded with SiPMs on the detectors’ surfaces. With the light pattern images as input, the network should predict the gamma-ray interaction positions.

#### Baseline method

In addition to our deep learning-based approach, we implement a simple centroiding-based method to serve as a baseline.

For every sample in the dataset, the baseline method performs the following steps to determine the scintillation position:

1. Compute the centroids of every light pattern with image moments.
2. Take the mean of the centroids of corresponding light patterns (top-bottom, left-right) to compute *x*-*z* and *x*-*y* centroids. This step is not applicable for the back light pattern as there is no corresponding front light pattern.
3. Take the mean of the corresponding dimensions of the *x*-*y*, *x*-*z*, and *y*-*z* centroids to compute the *x*-, *y*-, and *z*-centroid.
4. Stack the *x*-, *y*-, and *z*-centroid to create the predicted *x*-*y*-*z* position.

The predicted position is then compared to the ground truth value for every sample in the dataset to compute the MAE value of the baseline method.

#### Training pipeline

**Fig. 3:**
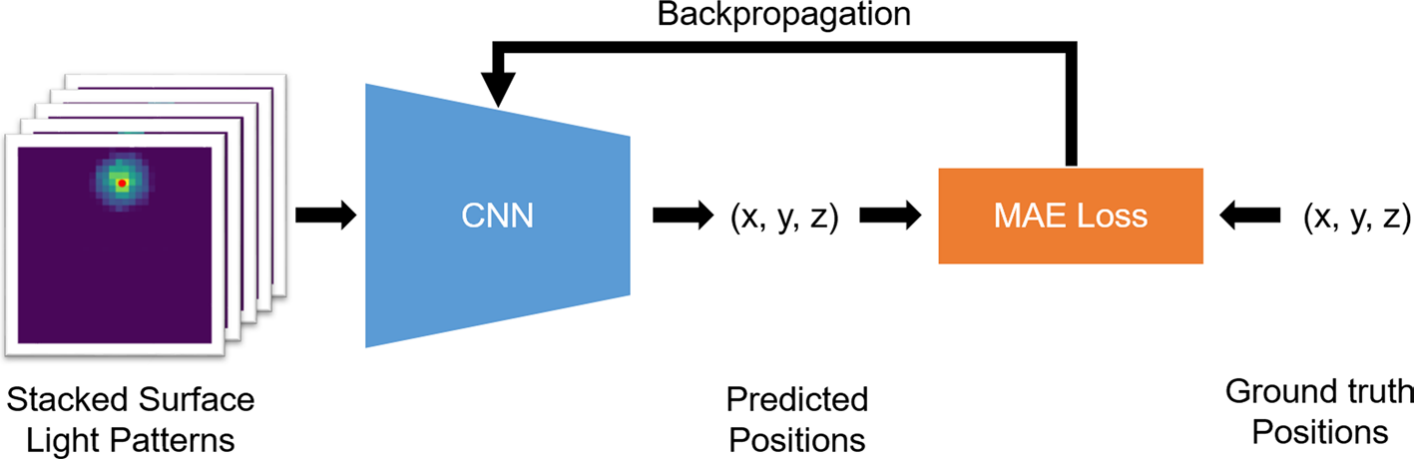
Training pipeline of the scintillation position prediction. The light patterns recorded on each surface are stacked along the channels dimension and used as input to the CNN. The CNN should predict the gamma-ray interaction position from the stacked light patterns. The MAE loss is computed with the predicted and ground truth positions and backpropagated through the network to update its weights

We implement the CNN training pipeline with PyTorch Lightning [24]. The code is organized with subclasses of the *LightningDataModule* and the *LightningModule*. In the LightningDataModule subclass, the train and test datasets are loaded, the train dataset is split into a train and a validation dataset, and the PyTorch *DataLoaders* are set up. In the LightningModule subclass, the network architecture, loss function, and optimizer are initialized. Additionally, the training, validation, and test steps are defined, including logging the loss and metrics. Weights & Biases [25] is used to create experiment sweeps and log results. The training pipeline of the scintillation position prediction is depicted in Fig. 3.

#### Experiment setup

In this work, the design of our proposed On-Chip PET system is optimized. We investigate the influences of the network architecture, crystal thickness, SiPM size, and the number of surfaces covered with SiPMs on the scintillation-position prediction performance. For the network architecture, we selected variants from the ConvNeXt [26], ResNet [27], and EfficientNet [28] families with different depths, as these architectures have shown great performances on image data. The parameters given in Table 7 are used for every experiment run. The experiments are performed on a machine with four NVIDIA GeForce RTX 3090 GPUs where one training takes approximately one hour to run.

**Table 7:** Parameters used for every experiment run

Parameter	Value
Dataset Splits	80k/800k train, 20k/200k val, 10k test
Input Tensor Size	Cx32x32/Cx24x24/Cx16x16
Network Architecture	ResNet18
Loss Function	Mean Absolute Error (MAE)
Optimizer	Adam [29]
Learning Rate	3e−4
Batch Size	256
Steps	50,000/500,000
Float Precision	16 bit

The number of input channels C is determined by the number of surfaces with SiPMs

### Sensitivity

We use the line source along the *z*-axis described in “Sources” section to determine the sensitivity of the system. The source has an activity of 1000 Bq and is simulated for 10 s. The sensitivity is computed by dividing the number of coincidence events where two interactions are recorded in two different detectors by the total number of events.

### Reconstruction

We evaluate the reconstruction performance of our proposed system with a grid of point sources and the more complex OOC phantom, as described in “Phantoms” and “Sources” sections.

For the reconstruction, it is necessary to be able to create LORs, which is only possible if the back-to-back gamma-rays interact in two detectors at the same time. For each of those sample pairs, the two corresponding scintillation positions are predicted with a trained network. All pairs of predicted scintillation positions are stored for further processing.

We perform the following steps for SART, an algebraic reconstruction method that shows good reconstruction performance in cases where there is limited data available [14]:

1. Load all predicted pairs of scintillation positions from disk.
2. Compute the distance and angle of the LOR to the origin for each LOR that is defined by the pair of predicted scintillation positions.
3. Create the sinogram from the LORs by computing the 2D histogram of the distances and angles with a bin size of 400 in both dimensions.
4. Generate the corresponding reconstructed image by running five iterations of SART implemented in scikit-image [30].

## Results

### Scintillation position prediction

#### Baseline method

The baseline method using a centroiding-based approach as described in “Baseline method” section achieves MAE values on the test dataset ranging from 2.72 mm for large SiPMs to 3.18 mm for small ones for a crystal thickness of 13 mm. The results for a crystal thickness of 26 mm are around 2.3 mm higher. Table 8 contains the prediction errors for all six combinations of crystal thicknesses and SiPM sizes.

**Table 8:** Scintillation-position prediction results of different SiPM sizes and crystal thicknesses of the baseline method

Crystal thickness [mm]	Small MAE [mm]	Medium MAE [mm]	Large MAE [mm]
13	3.18	3.12	2.72
26	5.46	5.43	5.40

The shown MAE values are computed on the test dataset

#### Crystal thickness and SiPM size

With the first set of CNN training runs, we determined the optimal crystal thickness and SiPM size. For the crystal thickness, we evaluate two options, 13 and 26 mm, as those could easily be realized in reality with stock SiPM arrays. For this set of experiment runs, the light patterns from all five surfaces covered with SiPMs are used as input to the network and the training datasets with 100k samples are used. Table 7 depicts the other training parameters used for these runs. Table 9 shows the prediction performances on the validation dataset. The MAE achieved with 13 mm thick crystals is 0.98 mm for the small SiPM size, 0.99 mm for the medium, and 1.00 mm for the large ones. The results for 26 mm thick crystals are around 0.20 mm worse for each SiPM size.

**Table 9:** Scintillation-position prediction results of different SiPM sizes and crystal thicknesses of the first set of experiment runs

Crystal thickness [mm]	Small MAE [mm]	Medium MAE [mm]	Large MAE [mm]
13	0.98	0.99	1.00
26	1.18	1.19	1.22

The shown MAE values are computed on the validation dataset

#### Network architecture

With the second set of CNN training runs, we determine the best working network architecture among variants from the ConvNeXt, ResNet, and EfficientNet families with different depths. For this set of experiment runs, the GATE simulation is run with a crystal thickness of 13 mm, the light patterns are created with the small SiPMs, the training dataset with one million samples is used, and the light patterns from all five surfaces covered with SiPMs are used as input to the network. Table 7 depicts the other training parameters used for these runs. Table 10 shows the prediction performances of the different network architectures on the validation dataset. ConvNeXt Tiny, ConvNeXt Large, and ConvNeXt Base are the architectures that perform best with an MAE value of 0.82 mm closely followed by ResNet50 with 0.83 mm and ResNet18 and ResNet101 with 0.84 mm. The three architectures from the EfficientNet family achieve results between 0.88 and 0.90 mm.

**Table 10:** Scintillation-position prediction results of different network architectures for the small SiPM size

Network architecture	Mean Absolute Error (MAE) [mm]
ConvNeXt Tiny	0.82
ConvNeXt Large	0.82
ConvNeXt Base	0.82
ResNet50	0.83
ResNet18	0.84
ResNet101	0.84
EfficientNet-B0	0.88
EfficientNet-B4	0.90
EfficientNet-B7	0.90

The shown MAE values are computed on the validation dataset

#### Combination of light patterns as input

With the third set of CNN training runs, we determined the optimal combination of input light patterns. All possible combinations of the five surfaces, in total 31, are evaluated for a crystal thickness of 13 mm, the training dataset with one million samples, and the small SiPM size. The training parameters are those from Table 7. Table 11 contains the prediction results. The best ten combinations achieve results close together with MAE values between 0.8237 mm and 0.8370 mm. The worst performances are achieved with the top and bottom surfaces with an MAE of around 3.9 mm.

**Table 11:** Scintillation-position prediction results of different surfaces as input

#	Surface count	Surfaces	Mean Absolute Error (MAE) [mm]
1	3	**ba-le-ri**	0.8237
2	3	ba-ri-to	0.8240
3	4	**ba-bo-le-to**	0.8245
4	4	ba-bo-le-ri	0.8270
5	5	**ba-bo-le-ri-to**	0.8270
6	4	ba-bo-ri-to	0.8281
7	3	ba-le-to	82.93
8	2	**ba-ri**	0.8309
9	1	**ba**	0.8370
10	3	ba-bo-ri	0.8370
...			
29	2	bo-to	1.475
30	1	to	3.764
31	1	bo	3.806

The best performing combination for every number of light patterns is shown in bold. The first two letters of each surface name are used as abbreviations for the surfaces: ba—back, bo—bottom, le—left, ri—right, to—top. The shown MAE values are computed on the validation dataset

#### Best result on test dataset

**Fig. 4:**
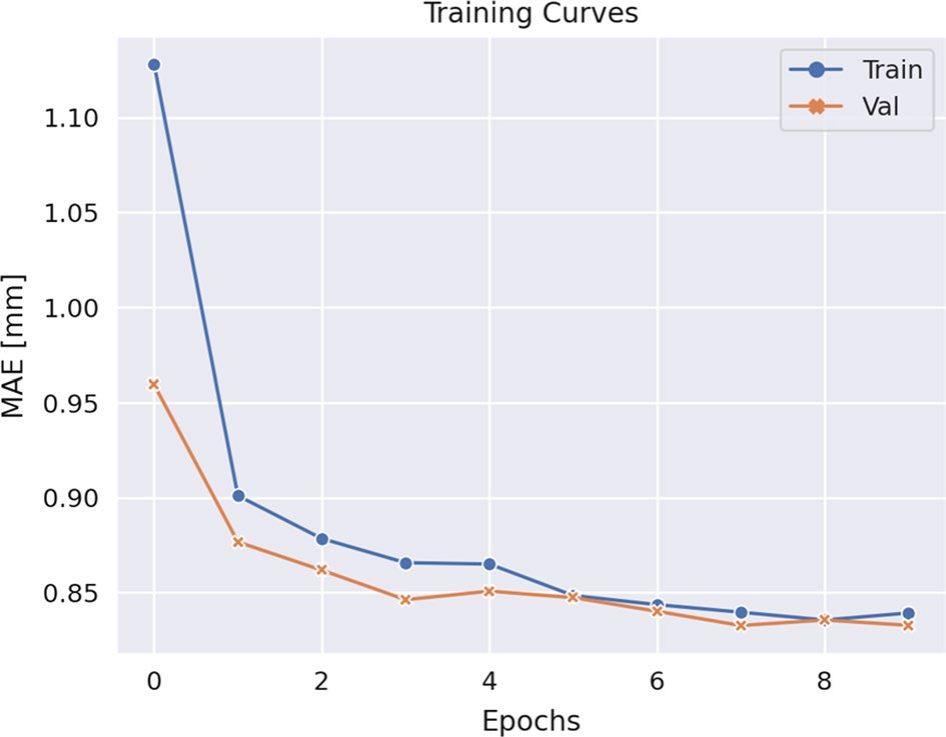
Training curves of the network with the best performing design choices. The train MAE is depicted in blue and the validation MAE in orange

We trained a network with the best-performing design choices from the three experiment runs—a crystal thickness of 13 mm, a SiPM size of 3 mm, the ConvNeXt Tiny architecture, and the back-left-right surface combination—with the training dataset consisting of one million samples. This network achieves an MAE of 0.80 mm on the test dataset and is used for the reconstruction pipelines described in the following section. The training and validation loss curves are shown in Fig. 4.

#### Anisotropy of positioning error

**Fig. 5:**
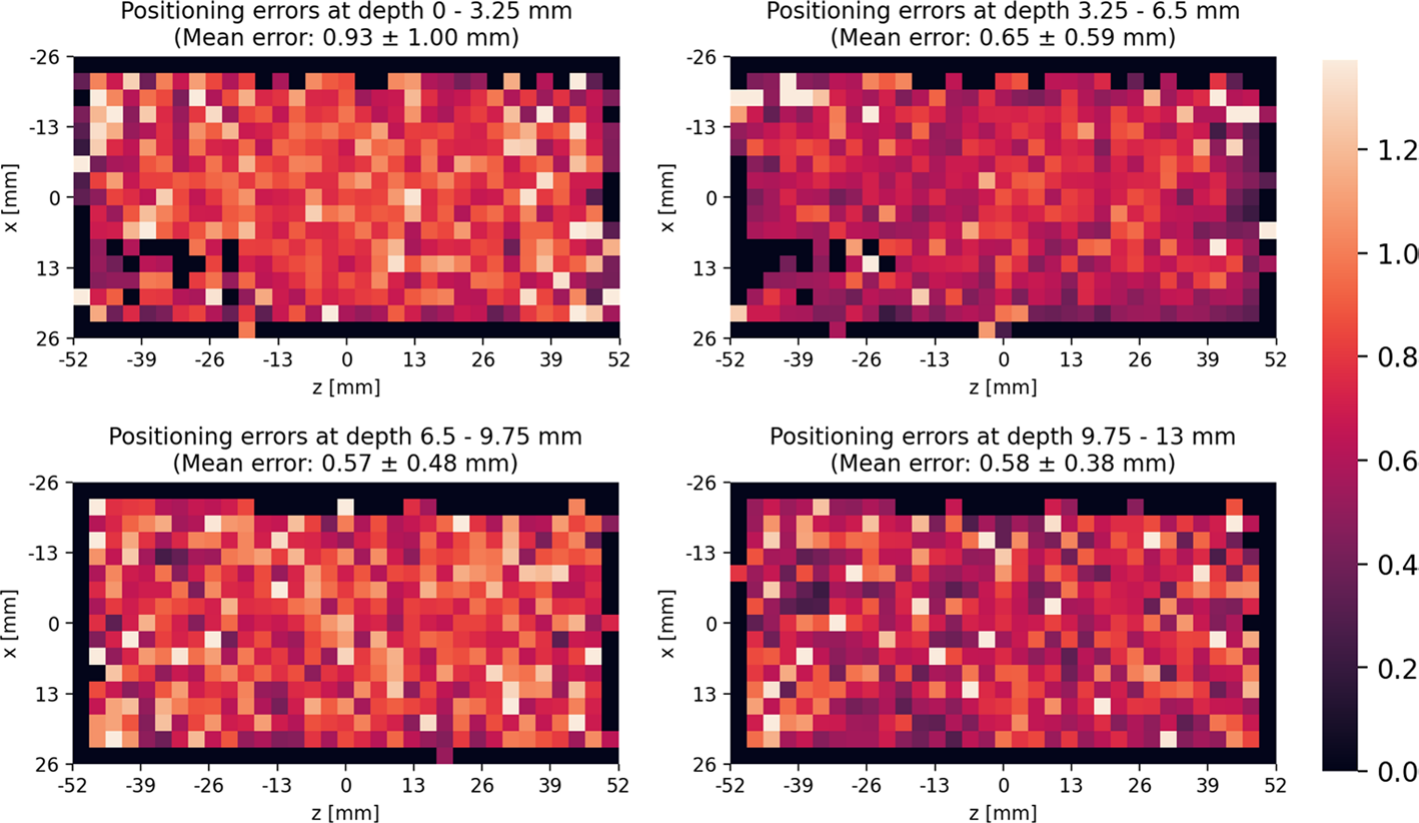
Anisotropy of the predicted interaction position at different crystal depths. The positioning error between the predicted and ground truth positions is plotted across the *x*-*z* dimensions for crystal depths between 0 mm and 3.25 mm (top-left), 3.25 mm and 6.5 mm (top-right), 6.5 and 9.75 mm (bottom-left), and 9.75 mm and 13 mm (bottom-right)

**Fig. 6:**
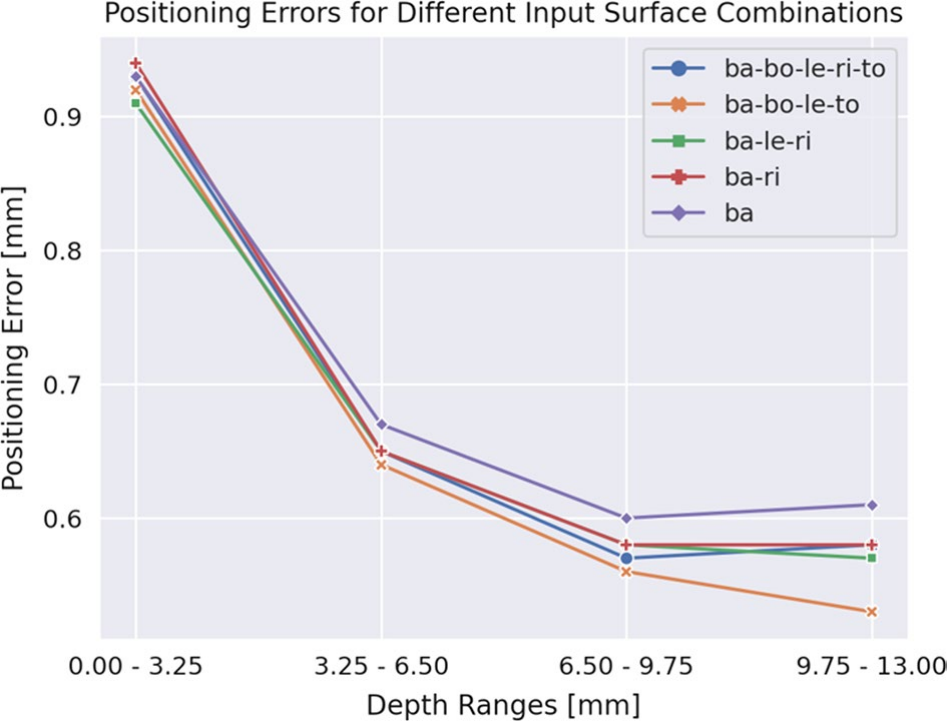
Positioning errors for different input surface combinations at depth ranges. The mean positioning error is computed for depth intervals of 3.25 mm. The best performing combination for every number of surface light pattern from Table 11 is used to predict the first interaction positions

Figure 5 shows the anisotropy of the scintillation-position error at different crystal depths. The network taking all five surfaces covered with SiPMs from Table 11 is used to predict the scintillation positions. Figure 6 depicts the positioning errors for different input surface combinations at depth ranges. The mean positioning error is computed for depth intervals of 3.25 mm. The best performing combination for every number of surface light patterns from Table 11 is used to predict the first interaction positions.

### Sensitivity

We recorded 3481 coincidence events from the line source simulation with 13 mm thick crystals where a total of 10,000 events were created and 4072 coincidence events for 26 mm thick crystals. For the 13 mm thick crystals, there we no random coincidence events, and for the 26 mm ones, there were four. This leaves us with sensitivities of 34.81% and 40.68% for the 13 mm and 26 mm thick crystals, respectively.

### Spatial resolution

**Fig. 7:**
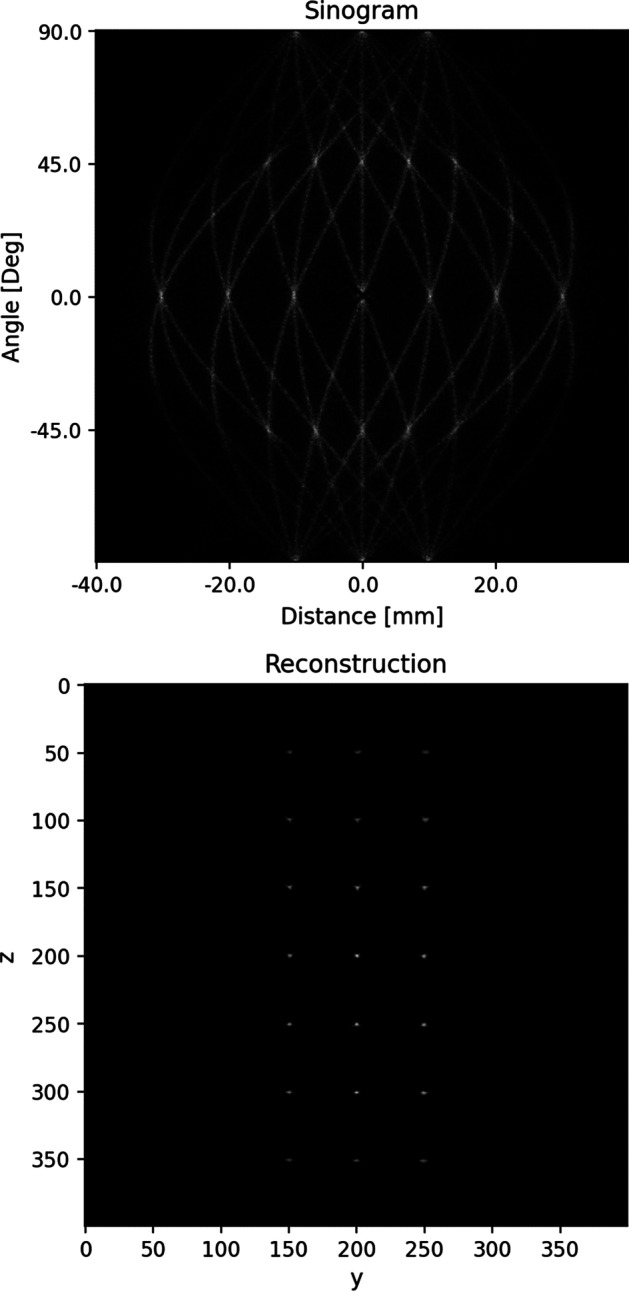
Sinogram and corresponding reconstructed image of point sources arranged in a 7 $\times$ 3 grid with a distance of 10 mm between each source. The best performing model from “Best result on test dataset” section is used to predict the scintillation positions. The reconstructed image is created with SART [14]

We use the best-performing model from “Best result on test dataset” section to predict the pairs of interaction positions of the point-sources grid dataset described in Table 5. The SART steps from “Reconstruction” section are performed to create the sinogram and reconstructed image shown in Fig. 7.

**Fig. 8:**
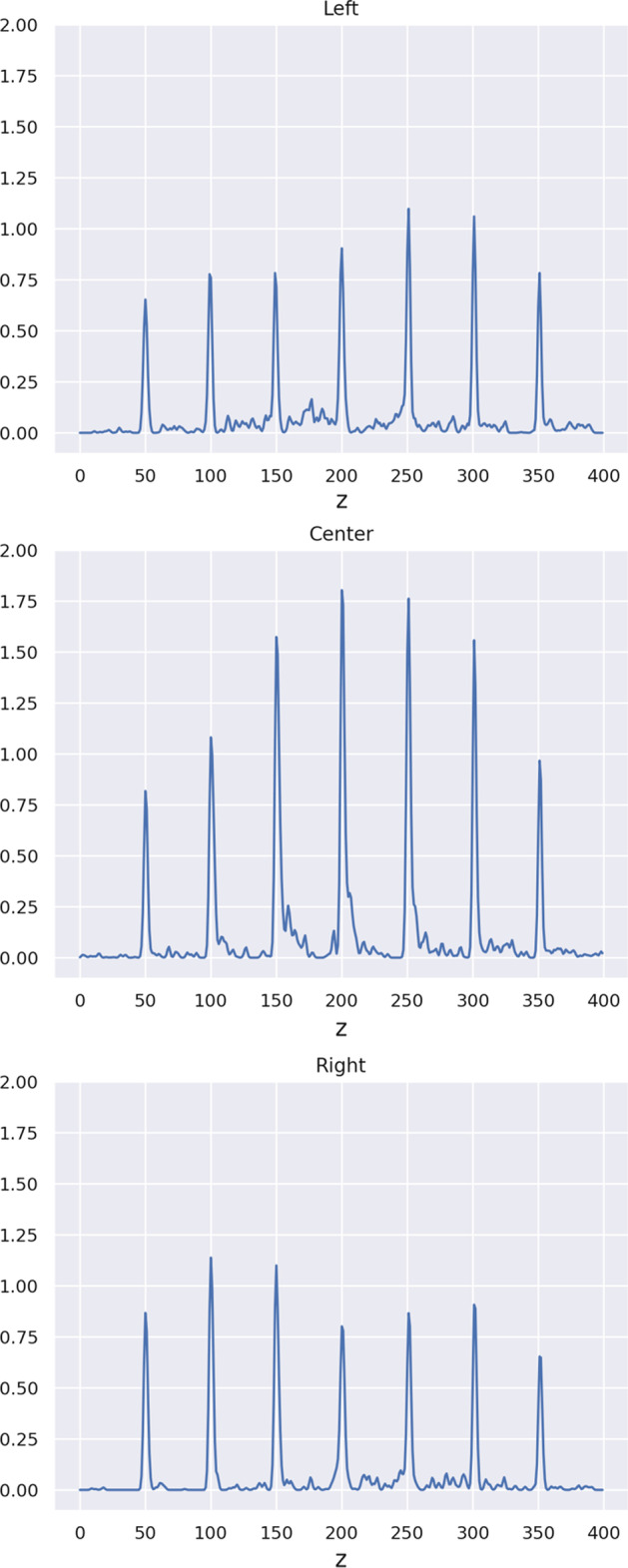
Z-Line profiles drawn through the reconstructed image Fig. 7. The profiles are Gaussian filtered with a sigma of 0.5

Table 12 depicts the spatial resolution FWHM values for each of the 21 point sources. The mean FWHM value is 0.55 mm with a standard deviation of 0.19 mm. The FWHM values are computed by averaging the peak half widths of *x*-, *y*-, and *z*-line profiles drawn through the reconstructed image. The profiles are Gaussian filtered with a sigma of 0.5. Figure 8 depicts the *z*-line profiles drawn through the reconstructed image from Fig. 7.

**Table 12:** Spatial resolution FWHM values in mm of the reconstructed image of the grid of point sources shown in Fig. 7

Row/Column	1	2	3
1	0.59	0.53	0.79
2	0.39	0.45	0.51
3	0.28	0.45	0.51
4	0.63	0.51	0.69
5	0.54	0.49	0.65
6	0.48	0.51	0.60
7	0.49	0.70	0.74

### OOC phantom reconstruction

**Fig. 9:**
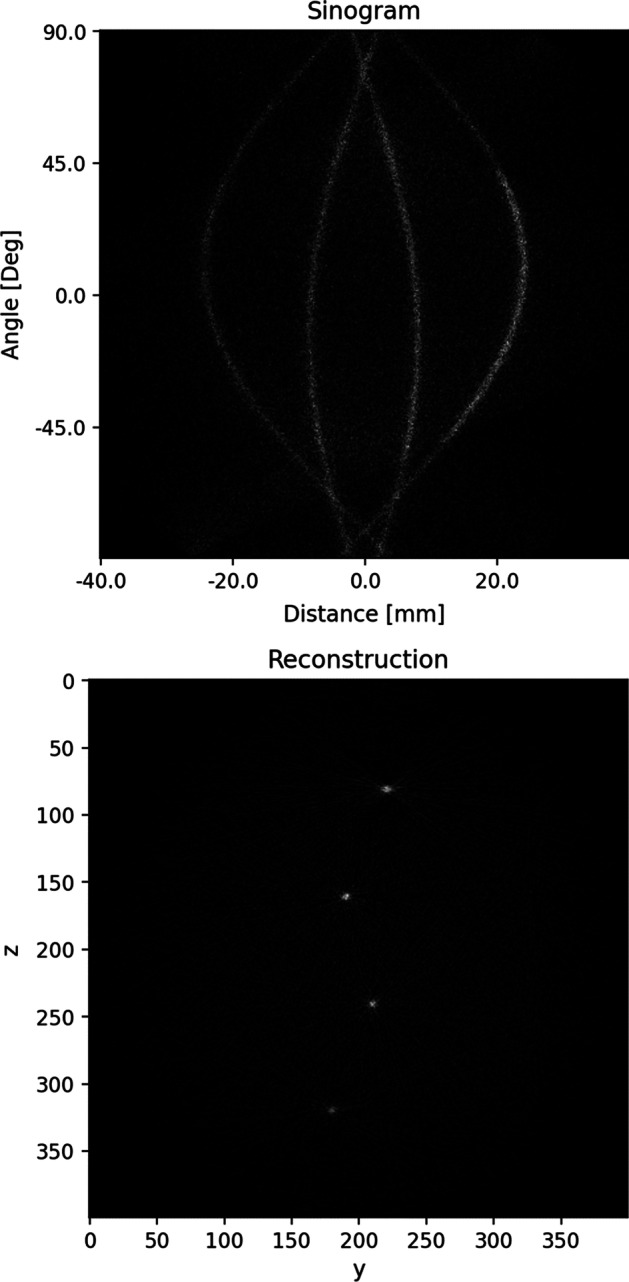
Sinogram and corresponding reconstructed image of the OOC phantom described in “Best result on test dataset” section. The phantom consists of four sphere-shaped hot sources with radii of 0.4 mm, 0.5 mm, 0.6 mm, and 0.7 mm. The hot sources are surrounded by sphere-shaped cold sources with a radius of 2 mm each. The activity concentration of the hot sources is 1000 Bq/mm$^3$ and 100 Bq/mm$^3$ for the cold sources

**Fig. 10:**
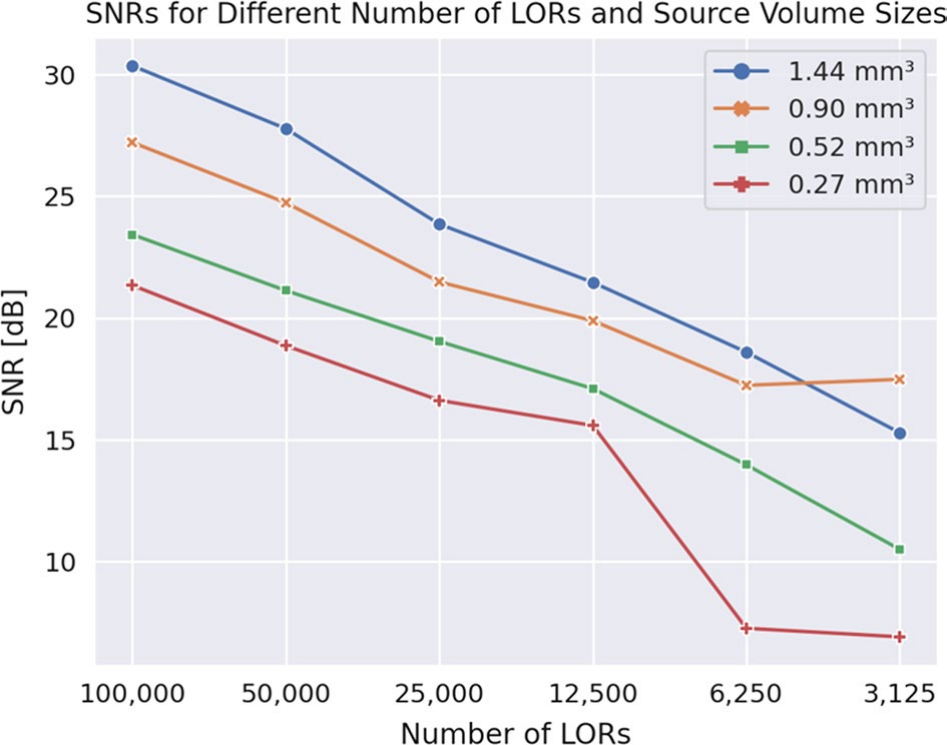
SNRs for different number of LORs and source-volume sizes. The OOC phantom described in “Phantoms” section is reconstructed with different numbers of LORs to determine the minimum number of LORs needed for an adequate image quality. The hot parts of the phantom are sphere-shaped volumes with radius ranging from 0.4 to 0.7 mm leading to the volume sizes used in the figure

We reconstruct images of the OOC phantom described section 2.1.2 in the same manner as the grid of point sources in the previous section. However, in this case, we perform multiple reconstructions with different numbers of LORs to determine the minimum number of LORs needed for an adequate image quality. Figure 9 shows the sinogram and corresponding reconstructed image where 100k LORs are used. In Fig. 10, the Signal-to-Noise Ratios (SNRs) for different numbers of LORs and source-volume sizes are shown. The SNRs values are computed by taking the mean from the *x*-, *y*-, and *z*-line profiles drawn through the center of each sphere source. The sphere-shaped source with a radius of 0.7 mm and a volume of 1.44 mm$^3$ achieves the best SNR of 30.36 dB when using 100k LORs. The SNR drop-off for the smallest source with a radius of 0.4 mm and a volume of 0.27 mm$^3$ when using 6250 and 3125 LORs comes from SNR values of 0 dB in one of the dimensions of the line profiles. The smallest SNR where there are three non-zero values from the line profiles is 10.49 dB achieved by the source with a radius of 0.5 mm and a volume of 0.52 mm$^3$.

## Discussion

### Influence of the crystal thickness and SiPM size on the prediction performance

The results from the baseline method in “Baseline method” section show an inverse relationship between the prediction error and the SiPM size: the larger the SiPM size, the smaller the MAE value. This can be explained as follows: the larger the size of the sensitive area of the SiPM, the more optical photons are detected by the SiPM, and therefore, the higher the output value of the SiPM. Thus, the maximum values of the light patterns created with larger SiPMs are higher, and the positions of the centroids are closer to the positions of the maxima. This in turn enables a better prediction performance, as the scintillation position correlates with the position of the maxima of the light patterns.

We implemented the baseline method to serve as a comparison to the deep learning-based approaches, which give almost one order of magnitude better results. The deep learning-based method in “Crystal thickness and SiPM size” section shows the opposite relationship compared to the baseline method between the prediction errors and the SiPM sizes: the smaller the SiPM size, the smaller the MAE value. This can be explained by the observation that CNNs perform better with increasing image dimensions. In our case, the light patterns created with small SiPMs have dimensions of 32 $\times$ 32, the medium ones 24 $\times$ 24, and the large ones 16 $\times$ 16.

When building an actual prototype of the system, we cannot simply choose the option with the best prediction performance but also have to take practical considerations into account. As Table 13 shows, the number of channels that need to be read out individually for one detector covered with small SiPMs is four times higher than when using large SiPMs, and 1.8 times higher compared when using medium SiPMs. At the same time, the MAE value for 13 mm thick crystals of the large SiPMs is only 2% higher than the one of the small SiPMs. Therefore, we need to find the right balance between system performance and technical feasibility as well as costs for the prototype.

**Table 13:** Number of channels for one detector for the different SiPM sizes

Surfaces	3 mm SiPM	4 mm SiPM	6 mm SiPM
Back	16 * 32 = 512	12 * 24 = 288	8 * 16 = 128
Left/right	8 * 32 = 256	6 * 24 = 144	4 * 16 = 64
Top/bottom	8 * 16 = 128	6 * 12 = 72	4 * 8 = 32
All five	1280	720	320

With regards to the crystal thickness, the design choice is easier to make. The results from the baseline method as well as the deep-learning based method show that the MAE values are around 20% higher for 26 mm thick crystals compared to 13 mm thick ones. Additionally, thinner crystals are also cheaper to manufacture. One downside of thinner crystals is their lower sensitivity compared to thicker ones.

### Influence of the network architecture on the prediction performance

The results from the network architecture runs described in “Network architecture” section show that the family of ConvNeXt networks is better suited to the task of predicting the first scintillation position than the one of ResNet and EfficientNet. Determining the best overall architecture is a close call, as the MAE values within the ConvNeXt family are quite close. The results for the three ConvNeXt variants lie within 0.0025 mm and are closely followed by ResNet50 and ResNet18. As we are dealing with relatively small images (32 $\times$ 32) compared and to increase the training and inference time of the network, it makes sense to choose the architecture with the smallest number of parameters, ConvNeXt Tiny.

### Influence of the number of input light patterns on the prediction performance

The results from the experiments with varying numbers of input light patterns described in “Combination of light patterns as input” section indicate that light patterns from certain surfaces encode significantly more information about the scintillation position than others. We observe that the back surface achieves a prediction error that is only 0.0133 mm higher than the best overall performance that uses three surfaces as input. The result from the back surface is also better than many combinations of two, three, and four surfaces. Another indication of the high amount of information that is encoded in the back surface is that the back surface is part of every combination of surfaces in the ten best results. This observation should be expected as the back surface is the largest of all surfaces and therefore should encode the most information about the scintillation position for the network.

From these results, we observe a further practical implication: we do not need to cover all five surfaces with SiPMs to achieve good scintillation-position prediction performances.

### Anisotropy of the predicted interaction positions

Figures 5 and 6 indicate that the positioning error decreases the deeper the scintillation takes place in the crystal. From Fig. 5 it is not clearly visible that the *x-* and *z*-positions of the scintillation influence the prediction performance. The heatmaps of the positioning errors do not show a degradation of the prediction performance near the boundaries of the detector as good (dark) and bad (light) spots are spread equally across the entire view. From Fig. 6 we observe that having SiPMs on additional surfaces instead of only on the back one increases the prediction performance more the closer the scintillation takes place to the back surface. From this figure, we also get an explanation of why the thicker crystals perform worse than the thinner ones. There is a clear relation visible between decreasing positioning error and increasing scintillation-position depth. The closer the scintillation takes place to the back surface the better the prediction performance. For the 26 mm thick crystals, the amount of scintillations that take place further away from the back surface is higher than for 13 mm thick crystals and therefore the prediction performance is worse for the thicker crystals.

### Spatial resolution

The SART reconstruction of the grid of point sources described in “Reconstruction” section shows good reconstruction performance over the entire OOC device. We observe that the FWHM values from Table 12 slightly drop off when moving from the middle row (4), where the best FWHM values are reached, to lower and higher rows. The worst results are achieved close to the upper and lower boundaries of the field of view of the scanner.

### OOC imaging capability

A qualitative evaluation of Fig. 9 lets us conclude that our proposed system is capable of imaging OOC devices containing volumes between 0.27 mm$^3$ and 1.44 mm$^3$. From Fig. 10, we observe a linear relationship between the SNRs and numbers of LORs for the four sphere sources with different volumes except for the drop-off described in section 3.4.

If we set the SNR threshold for an acceptable reconstructed image to above 16 dB, 25,000 LORs are needed to be able to reconstruct all four sphere sources with adequate quality. With an activity concentration of 1000 Bq/mm$^3$ in the hot regions (four spheres with different radii) and 100 Bq/mm$^3$ in the cold regions (four spheres with radii of 2 mm each), there is a total activity of 16,537 Bq in the system. With a sensitivity of 34.81%, around 73,500 events are needed for 25,000 LORs, which equals a recording time of fewer than 5 seconds. This means that it would be possible to perform pharmacokinetic analyses of OOCs with our proposed system, similar to the work of Liu et al. [13] with a higher resolution and smaller time interval. With the growing use of 3D models in radiopharmaceutical research, our system would enable deeper analyses of these models and provide a further tool for radiopharmacists to develop radiotheranostics [31].

## Conclusion

In this work, we introduced the concept of an On-Chip PET system that makes OOC imaging possible. The main challenge to overcome for PET systems in this task is their limited spatial resolution, which lies in the range of slightly more than 1 mm. Previous works have shown that it is possible to achieve resolutions of less than 1 mm with setups consisting of a monolithic crystal combined with advanced data-analysis methods using deep learning-based approaches.

In this work, we designed a system consisting of four detectors each made up of two monolithic LYSO crystals with SiPMs attached to multiple surfaces. We generated training, testing, and reconstruction datasets with a MCS of the system and observed that the ConvNeXt Tiny architecture achieved the best scintillation-position prediction results with a MAE value of 0.80 mm on the test dataset. The proposed system achieves a sensitivity of 34.81% for 13 mm thick crystals and 40.68 % for 26 mm thick ones. It does not show a interaction-position prediction degradation near the boundaries of the detector. With the trained network, we reconstructed a grid of point sources using SART and reached a mean FWHM value of 0.55 mm, which is close to the lower bound of PET spatial resolution without positron range correction.

The results from the scintillation-position prediction and reconstruction demonstrate the capability of our system to achieve a resolution of almost 0.5 mm for a large field-of-view using out-of-the-box reconstruction methods. We showed that it is possible to reconstruct the small volumes found on OOC devices and that our proposed system would be able to perform pharmacokinetic analyses of OOCs. As next steps, we will shift our focus from interaction-position prediction to reconstruction to achieve an even better resolution. We will develop a list mode-based reconstruction method incorporating the geometrical priors that our system and OOC devices are constrained by.

One limitation of the proposed system in its current state is that it would not be able to differentiate signals coming from an individual organoids. However, it would still be possible to capture the spread of the fluid inside the vessel system and measure uptake differences between different compartments on the OOC device. This would enable pharmacokinetics analysis of OOCs for disease modeling and precision medicine applications.

Our results showed practical implications that play a crucial role in the next project steps, where we are going to build a prototype of the proposed system. We compared the performances of different SiPM sizes and observed that using larger SiPM sizes results in a slight decrease in prediction performance only while reducing the number of channels that need to be read out individually. We also saw that using thinner crystals is advantageous and that the back surface encodes significantly more information about the scintillation position compared to other surfaces. Therefore, we concluded that not all five surfaces of each detector need to be covered with SiPMs. This reduces the number of channels that need to be read out individually further.

## Data Availability

The code and datasets used and/or analysed during the current study are available from the corresponding author on reasonable request.
